# Strengthening supervisor support for employees with common mental health problems: developing a workplace intervention using intervention mapping

**DOI:** 10.1186/s12889-022-13545-7

**Published:** 2022-06-08

**Authors:** Suzanne G. M. van Hees, Bouwine E. Carlier, Roland W. B. Blonk, Shirley Oomens

**Affiliations:** 1 Occupation and Health Research Group, HAN University of Applied Sciences, P.O. Box 6960, 6503 GL Nijmegen, The Netherlands; 2 Tilburg School of Social and Behavioral Sciences, Tilburg University, Tilburg, the Netherlands; 3 TNO, Leiden, The Netherlands; 4 Optentia, North West University, Vanderbijlpark, South Africa; 5 Department of Primary and Community Care, Nijmegen School of Occupational Health, Radboudumc, Nijmegen, The Netherlands

**Keywords:** Leadership, Supervisor support, Mental health, Staying at work, Intervention mapping, Workplace interventions, Organizational intervention, Occupational health, Prevention, Absenteeism

## Abstract

**Background:**

This study presents the development of a workplace intervention to strengthen supervisor’s support for employees with common mental health problems (CMHP). CMHP have been increasing over the last years, resulting into negative work outcomes, such as absenteeism or reduced work performance. To date, organisational interventions have been promising in preventing these negative work outcomes, however it is yet unknown in what way the role of workplace stakeholders, in particular supervisors, can be strengthened. This study contributes to the literature of interventions on an organizational level which uses a preventative approach by promoting stay at work among employees with CMHP through supervisor support.

**Methods:**

we applied the intervention mapping (IM) approach, by actively involving workplace stakeholders (employees with CMHP, supervisors and occupational health professionals) through the development process and the use of Integrated model of behaviour prediction for employers. All six steps of IM are followed and thematic analysis was used to analyse interviews and focus groups.

**Results:**

Based on a comprehensive needs assessment, the intervention resulted in an online guideline, with five step-wise themes on how to support employees with CMHP to stay at work (SAW). The guideline addressed the most important and changeable actions using the Integrated model of behaviour prediction. The guideline presents how to signal and address problems in the workplace and find solutions by stimulating autonomy of employees, explore job accommodations and ask for occupational support. In addition, basic conditions on how to create mentally healthy workplaces were presented. Coaching sessions by occupational health professionals, that include practical strategies using the best available evidence, were identified by the stakeholders.

**Conclusions:**

This SAW-Supervisor Guideline-intervention responds to the need of supervisors to be supported in their role, responsibility and ways to support employees with mental health issues, through a behaviour-oriented, preventative approach. Intervention mapping provided a systematic process to identify, structure and prioritize factors of supervisor support, resulting in a novel workplace intervention. The active involvement of workplace stakeholders throughout the process resulted into a well-received intervention. The theoretical framework provided practical ways to induce supportive behaviour of supervisors, bridging theory with practice.

## Background

To a greater or a lesser extent, everyone has to deal with mental health issues in life. At any point in time, one-sixth of the working age population is suffering from common mental disorders [1, 2]. Despite all efforts regarding preventative mental health interventions, the OECD and occupational health researchers call for more attention to employees with common mental health problems (CMHP) in the work context [1, 3, 4]. Work is often considered as an important cause of CMHP, and at the same time an essential solution to enhance mental health, societal participation and general wellbeing of individuals. Staying at work (SAW) while facing mental health issues can be used as a means to decrease the severity of CMHP, resulting in prevention of negative work outcomes such as absenteeism or reduced work performance for employees with CMHP [5]. Workplace stakeholders, especially supervisors, play a key role in prevention by supporting employees with CMHP, that may avoid employees with CMHP getting absent in the long term [6, 7]. We define SAW as to continue working while maintaining work performance [5]. Common mental disorders refer to depression, anxiety disorder, or stress-related disorder [8, 9]. However, a large number of employees who suffer from common mental health problems are undiagnosed and do not receive treatment, or do not disclose their diagnosis at the workplace [3, 10]. Therefore, we target a relatively broad group of employees with diagnosed mood, anxiety or stress-related problems as well as self-reported psychological complaints.

The literature in occupational health shows that high quality leadership predicted a reduced risk of long-term sickness absence [11] and contributes to return to work [12]. Various studies show how low supervisor support is a risk factor for absenteeism [13–15] and how investing in supervisor support, e.g. to facilitate the dialogue between employee and the nearest supervisor by following a protocol, contributes to better return to work planning [16]. Only a few studies show promising results that supervisor support enhances employees to stay at work because it is harder to know what worked in prevention of negative working outcomes, such as we aim in this study [5]. However, a trustful relationship with the supervisor, with whom the employee can discuss needed support or job accommodations, is found to promote SAW [5]. The increasing number of absenteeism and incapacity for work because of mental health problems over the last decades shows that it is challenging to intervene effectively in the phase of being at work, where practical guidelines for workplace stakeholders such as supervisors are scarce [17, 18]. This is urgent, because it is often the supervisor, their line manager, who is the first person who needs to act when the employee struggles at work. This workplace stakeholder is often not trained on how to do so accordingly [19]. In sum, research shows the important role that supervisors have in supporting these employees to SAW, however in case of CMHP they lack strategies or guidelines on how to support [19–21]. To illustrate, 40% of a representative panel of Dutch employers reported not to know how to help employees with CMHP in the workplace [22]. Therefore, there is a need to provide supervisors with clear directions on ways to promote SAW among employees with CMHP.

There are various reasons why the role of the supervisor in the phase of staying at work with CMHP is under addressed. First, although policies are into place on sustainable employment and promotion of health and wellbeing of employees, in practice, supervisors often act when the employee is yet facing reduced performance or sickness absence [23]. Second, signalling is hard because employees find it difficult to disclose mental health issues at the workplace, making it harder for supervisors to address mental health [24]. Third, CMHP usually develop slowly and saliently. Altogether, talking about mental health at the workplace is frequently avoided by both employees and supervisors due to the stigma and fear for losing the job [25]. In the Netherlands, due to privacy laws, supervisors are not allowed to ask or even know about the employee’s medical condition. Altogether, it is complex for supervisors to effectively support and facilitate employees due to the lack of guidance on their role and ways to deal with mental health in the workplace. This study aims to develop such an intervention, to strengthen supervisor support for employees with CMHP, derived from research and practice.

Well-designed work and workplaces that promote SAW seem essential to prevent negative work outcomes [2]. For this, effective, preventive workplace interventions are needed. Although organizational interventions have been shown promising in preventing mental health problems of employees [26, 27], it is yet unknown what the elements and effects of such interventions are on actual supervisors’ supportive behaviour [7, 28]. So far, preventive interventions that target supervisors’ behaviour as a mechanism of change in employee health, well-being and work outcomes consist of elements such as a behaviour oriented approach [28, 29] and a participative problem solving approach [30]. A supportive supervisor can open the door for employees with CMHP regarding their needs for organizational support, e.g. by offering job accommodations or time for treatment. Therefore, it would be valuable to investigate what in the behaviour of supervisors works or does not work to promote SAW for employees with CMHP. Because it is harder to investigate effects of what has not yet occurred, such as in prevention, [23], it is challenging to know for both employees and their supervisors what can be done in the workplace through a preventative approach [3]. Relatively few studies are specifically investigating the role of supervisors in prevention, in order to support employees with CMHP to SAW. Therefore, we need to explore what happens in practice and use those learned lessons to develop interventions [21].

Previous studies targeted supervisor support to reduce negative work outcomes for various employee populations. One promising intervention was presented in a study targeting self-efficacy of supervisors based on the ASE model [31], aiming to reduce negative work outcomes. This study used strategies such as inter-collegial consultation [32]. Other studies used different theoretical frameworks, two using the Self-determination theory [33, 34] and one using the trans-theoretical framework [35], offering more insights into the behavioural elements of workplace stakeholders. To create mentally healthy workplaces, we assume, as those studies, that it is necessary to target individual behaviour of various workplace stakeholders [2, 6, 36]. In addition, we emphasize the importance of workplace factors on organisational level. In the previous intervention studies, it remained unclear how environmental factors, such as the learning climate or social safety were targeted or evaluated. Therefore, in the present study, we used the Integrated model of behaviour prediction to frame employer’s behaviour that also incorporates environmental factors [37].

Besides, the use of a practical, participative approach to intervene is needed. A protocol providing insights and transparency based on theory and evidence may provide support on the development of such an intervention. We searched a systematic approach, in which Intervention mapping (IM) [38] has been applied previously in workplace interventions. However, it was most often used to target behaviour on the individual level for specific working populations [33, 39, 40]. Two studies applied IM on behaviour of workplace stakeholders such as supervisors [35] or occupational health physicians [34], however not on the promotion of Stay at work for employees with CMHP. This study aims to present the development of such an evidence-based workplace intervention. To meet the recommendations of recent reviews on the use of IM in workplace interventions [41, 42], we present how active stakeholder involvement, and the use of a theoretical framework were applied to bridge the gap between theory and practice.

## Methods

### Procedures

**Fig. 1:**
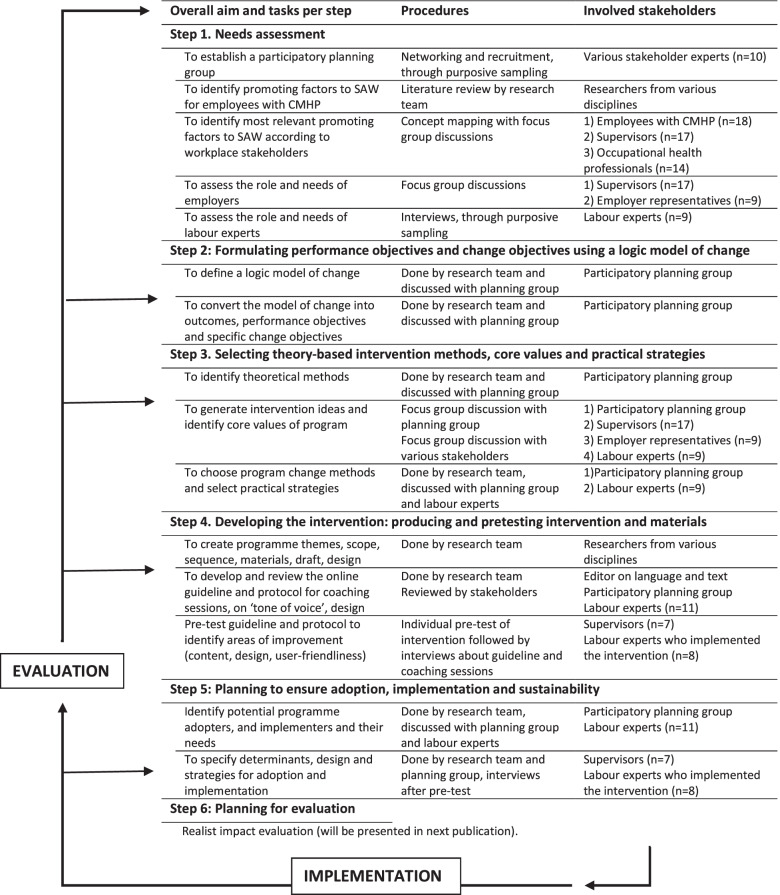
Intervention mapping process for development of the intervention. Legend: Overview of each IM step: overarching aims(s), tasks, procedures and stakeholders involved in the development of the SAW-SG intervention. Figure based on Intervention Mapping as described by Bartholomew [38]

This paper describes the development of the Stay at Work-Supervisor Guideline (SAW-SG) intervention (Fig. 1). This process was guided by the six steps of the IM approach for development, implementation and evaluation of health promotion interventions [38]. IM consists of six consecutive steps: 1) needs assessment, 2) formulating outcomes and intervention objectives using a logic model of change, 3) selecting core values, methods and practical strategies, 4) developing the intervention, 5) planning for adoption and implementation, and 6) planning for evaluation. IM is a stepwise process, and each step is based on previous steps. This study has been approved by the Ethical Review Board of Tilburg University, The Netherlands (EC-2019–30 and RP281).

### Selection of participants

In each step several workplace stakeholders were involved: supervisors, employees with CMHP and occupational health professionals (OHP), see Fig. 1. In this study we involved OHPs who are trained as so called “labour experts” in the Dutch social security system. These professionals are expert in the assessment and interventions needed in return to work processes, matching the employee’s capabilities with work and work environment. In order to include each workplace stakeholder group, purposive sampling was applied for recruiting participants. Purposive sampling allows for selection based on a sampling frame aiming to maintain rigor and identify based on specific study driven variables or characteristics [43, 44]. For each stakeholder group, participants were selected with respect to gender, age, working experience (varying from 1 to over 25 years of supervisory experience), size of organization (including medium and small sized organizations) and various sectors. Thereby, all participants were recruited in various ways, through the researchers’ network, promotion on websites of the national association for employers or labour experts and advocacy organizations representing people with CMHP, social media (LinkedIn) and existing expert groups. Participating OHPs were employed in various organisations (public and private) or self-employed and hired by organisations. Supervisors were working in sectors such as health care, IT, education, and civil services. Included supervisors reported to have dealt with employees facing CMHP at the moment or recently, based on their self-report. For the concept mapping study in step 1, we refer to the concept mapping study, regarding the selection of participants and data collection [31].

### Data collection

At the start of this study, a participatory planning group was established. The planning group consisted of occupational health professionals, representatives of employees with CMHP, representatives of the Dutch employers’ association, supervisors and researchers. Meetings were held regularly (half-yearly) to collect information as well as report on the progress and output of the steps throughout the IM process. These meetings were drafted in a way that participants received output of steps on the intervention development or intermediate results and were asked, through group discussion, to reflect upon this. Also, we held brainstorm sessions on preparation of the next IM step.

In each step of the IM process we collected data with relevant stakeholders. In step 1, needs assessment, we used two data collection activities, one was a concept mapping study, published elsewhere to explores perspectives of employees with common mental health problems, supervisors and OHPs on factors that promote SAW and its relative importance. Clustering those statements and scoring the relative importance led to concept maps in which stakeholders had various clusters in common. In the other part, through focus group and interviews, we collected data regarding the needs (step 1) and the subsequent IM steps, getting information during live and online interactive sessions on the development of the intervention to strengthen supervisor support. Four focus groups and 17 interviews were held by using group discussion, brainstorm techniques such as mind map, individual interviews asking feedback on prepared materials such as the guideline and group reflection on the developed intervention materials. Discussed topics were about their needs, including organizational needs, roles of workplace stakeholders, preferences regarding interventions to strengthen supervisor support, and regarding the particular characteristics of such a workplace intervention. All participants signed informed consent before participation. All of the focus groups and interviews during the IM study were audiotaped and transcribed verbatim. Thematic analysis was conducted by two researchers who independently coded relevant text fragments and labelled into categories [43]. Thereafter the researchers compared themes, to synthesize the results into general recommendations. In case of disagreement, topics were discussed by the research team until consensus was reached. The study took place in 2019 (step 1), 2020 (step 2, 3 and 4) and 2021 (step 5 and 6).

## Results

### Step 1. Needs assessment

#### Literature review

The literature review consisted of a realist synthesis that revealed what works to promote SAW among employees with CMHP, for whom, under what circumstances and how. The results of the review have been published elsewhere [5]. In sum, the synthesis, including 61 studies, demonstrates how a safe organisational climate and social support, especially by the supervisor, enable employees with CMHP to stay at work. More specifically, a trustful relationship in which the supervisor shows openness to talk about mental health conditions in an open climate, contributes to stay at work. Adequate and timely social support, from colleagues but particularly supervisors who are willing to assist and listen to work-related problems, increase the chance to stay at work among employees with CMHP. It was supposed that employees with CMHP can realize to stay at work through the following set of capabilities: a) by having meaningful relations and social support at work, b) by exerting control, c) by evaluating and adjusting the workload, d) by experiencing freedom to create opportunities for active coping, e) by experiencing better health, increased cognitive functioning and work performance. Facilitation, by an OHP, who acts independently, with sympathy and pragmatism, who provides an expert insight and who is familiar with the work and the work environment, also improves the likelihood to stay at work.

The literature review showed that most interventions still intervene on the individual, employee-level. The synthesis found that if those interventions focus on multiple elements, for example addressing both personal factors (symptom reduction and coping with symptoms) and work factors (coping at the workplace or a better work-related health), this leads to an increased likelihood to stay at work. Also, combining different strategies in interventions seemed necessary to change behaviour, such as an online guideline combined with the dialogue with a professional and homework assignments [5]. The results of this review were used to frame elements to promote SAW more thoroughly and provide content for the intervention.

#### Concept mapping study with multiple workplace stakeholders

For this study, workplace stakeholders (employees with CMHP [*n* = 18), supervisors (*n* = 17) and OHP (*n* = 14) provided statements on the focus question “What an employee with mental health problems needs to stay at work is…”.

First, participants emphasized on the role and needs of the employee in this phase of being at work while facing mental health issues. Unambiguously, it is significant for employees with CMHP to experience a sense of autonomy and meaning in work, even when struggling at work. Especially in this phase, it deemed important to experience self-control in work and a sense of responsibility to address problems. Participants mentioned it is important for them to jointly consider solutions, in which both employee and employer take their responsibility to act and intervene.

Second, supervisor support, reflected by a trustful relationship and empathic communication, is perceived to be highly important because it enables employees to address problems. A pre-existing strong work relationship, that is based on trust, sincere interest, openness and transparency is crucial to adequately support employees who struggle at work, because it encourages employees to earlier disclose and converse about their mental health problems. In that last case, this dialogue between employee and supervisor is ideally held in a social safe work environment. Such an environment enables them to discuss the impact of problems in work and what the employee needs to stay at work, ideally with an involved supervisor who adheres a pro-active, open, listening and non-judgmental attitude.

This leads to the third point, that work should be matched to the employee’s capabilities and needs through (timely and temporarily) work- or workplace accommodations. Also, professional and organizational support should be arranged by the employer. It was emphasized that the employee and supervisor should be in contact regularly, to assess and monitor the tailored job accommodations or interventions. Lastly, the occupational health service provider and the organization should set a clear goal, based on a shared vision on how to promote SAW and should collaborate to select tailored interventions in a particular case.

#### Focus groups and interviews with supervisors and OHPs

In addition to the above findings in which we investigated promoting factors to SAW for employees with CMHP, we also explored the needs of supervisors and OHPs on how to support employees to SAW through interviews and focus groups.

Table 1 summarizes the most important findings of the needs of supervisors in order to promote SAW, in random order. In sum, supervisors expressed that they need to be facilitated by their own organization in coaching or tools to gain knowledge. Also, they need skills on conversing about mental health and work. They especially lack knowhow on early signalling of mental health problems and information on what they can or cannot ask the employee. Besides, they express the need to know what interventions and job accommodations to offer and how to communicate about this accordingly, both towards the employee as towards the rest of the team. Having easy access to an OHP for consultation or being trained by them was given as a solution by supervisors.

**Table 1:** Step 1: summary of needs assessment

Focus group discussions
**Supervisor needs to support Stay at work (SAW)**
- Facilitation (given time to spend on intervention) from own organization (higher management, HR)
- Conversational skills training on mental health and work
- Safe working climate and openness to discuss mental health with employee without interference or effect on performance assessments or contracts
- Information about rules and regulation on prevention of sick leave and on roles and responsibilities of themselves, OHP and employees and information about boundaries where to hand over to OHP or another expert
- Knowledge and skills on interventions to offer (internal and/or external), in order to support SAW
**Preferences in an intervention**
- Addresses employee’s needs (self-control in work, sense of responsibility to address problems, matching and evaluating work, freedom to create opportunities for active coping, tailored work accommodations and interventions.)
- Easy access and strong collaboration with OHP, by receiving advice/consultation on a case or a coach
- Autonomy as supervisor for a tailored approach or exceptions
- Coaching to increase knowledge and positive attitudes of supervisors towards diversity and mental health
- Guidelines with practical tools and actions (tips & tricks)

Supervisors mentioned they prefer practical tips when being coached and a tailored approach during the intervention. Supervisors emphasized that such coaching could increase knowledge and positive attitudes towards diversity and mental health, needed to be reflected by all layers of their organization. They said to prefer easy access and strong collaboration by an expert in occupational health, also in this preventative phase. OHPs confirmed that in order to discuss ideas, share knowledge and increase skills of supervisors, they will need a guideline as a “conversation tool”, including a protocol on how to implement this intervention.

From this comprehensive needs assessment, we conclude that strengthening the individual supportive behaviour of the supervisor seems crucial to promote SAW among employees who struggle but stay at work. More specifically, we hypothesize that intervening on strengthening behavioural determinants of supervisors (e.g. attitude, skills, self-efficacy) will lead to supportive behaviour, which in turn might enable employees with CMHP to (partly) stay at work. Furthermore, the needs assessment revealed that supervisors can only effectually signal mental health issues and support employees with CMHP in a safe organizational climate. Having strong work relations among team members and supervisor and to know employee’s regular working behaviour seems conditional for a “mentally healthy workplace”. Supervisors expressed a need to be strengthened through coaching in ways to promote SAW among these employees.

### Step 2: outcomes and objectives using a logic model of change

#### Program outcomes

**Fig. 2:**
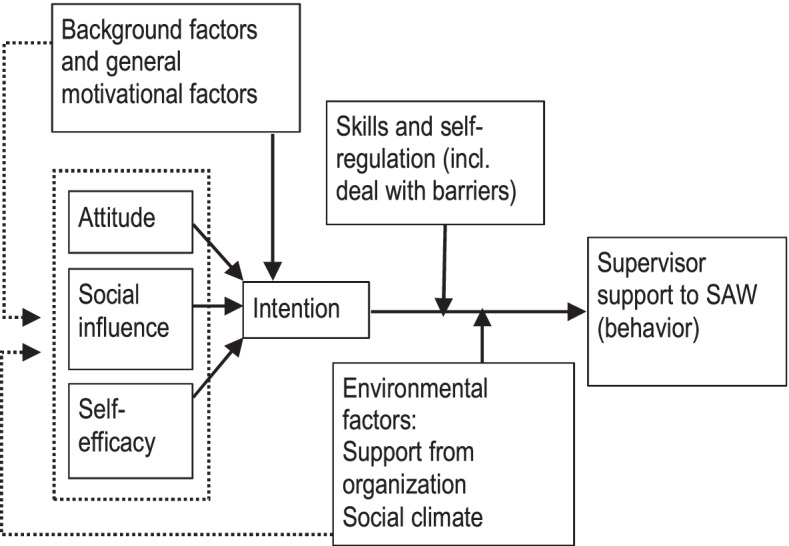
Step 2: logic model of change: Integrated model of behaviour prediction, applied to supportive behaviour of supervisors

In the second step, together with the planning group, a specific logic model of change was developed including program outcomes and objectives. The model was chosen based on the literature and group discussion on the feasibility of this model in the study context. The Integrated model of behaviour prediction for employers [37], depicted in Fig. 2, is selected as the logic model of change. This model assumes that the supervisors’ individual behaviour is based on their *skills*, as well as on their *intention*, influenced by attitudes, social pressure, self-efficacy and general motivational factors. This model incorporates general motivational factors and environmental factors, that permits in our model of change to adhere an integrative approach with the work environment. It also adds Bandura’s notion of self-efficacy, and intention, attitude and social norms, as an extension of the reasoned action approach [31]. Reflecting on the results of the needs assessment, those behavioural determinants match well, as it is important to have positive attitudes and social influences towards mental health at the workplace and believe in themselves (self-efficacy) to signal and address problems with employees. Supervisors may need to increase skills on how to deal with problems at work due to CMHP. Besides, this model integrates environmental (organizational) factors that influence the behaviour of supervisors. These reflect for example organizational support, team responsibility, the role of higher management, or the learning climate within the organization. Prioritization occurred by selecting the most relevant and changeable actions for supervisors, so these environmental, mostly contextual factors are therefore considered as basic conditions and not addressed as outcomes of the intervention. Criteria to select these actions were that actions were work-related, prioritized as important in the concept mapping study and selected on relevance and changeable by SV and OHP in the focus group sessions.

In conclusion, supervisor’s support outcomes were defined as follows:

- The supervisor’s intention to support, which may be influenced by general motivational factors, attitude, social influence, self-efficacy;
- The supervisor’s skills to support and how to deal with problems at work;
- The supervisor’s actual supportive behaviour, which may be influenced by the intention, skills and environmental factors.

#### Performance objectives and change objectives

The main objective of the intervention is to strengthen the supervisor’s supportive behaviour to promote staying at work for employees with CMHP. A large variety of behavioural actions were mentioned, resulted from the needs assessment. Subsequently, the abovementioned behavioural outcomes were operationalized into a sequence of actions, clustered into five performance objectives (Table 2). These five performance objectives are based on the “employee’s journey”: from having no problems in work to being on short term sick leave due to CMHP. Translating the performance objectives into more specific change objectives involved a thorough and rigor selection of behavioural determinants. A matrix of these change objectives was developed. Table 3 provides three examples of change objectives per determinant, aiming to define what the supervisor has to learn or change in order to perform the specific behaviour [42]. Full matrices are available upon request.

**Table 2:** Step 2: performance objectives

Performance objectives to promote Stay at work for employees with CMHP
1. Supervisor learns the signals and risk factors of CMHP and the impact of early signalling on work outcomes
2. Supervisor is able to talk with employee about the way CMHP affect one’s work
3. Supervisor is able to stimulate employee’s autonomy and sense of responsibility once MHP affects work
4. Supervisor learns to explore, facilitate and regularly evaluate job accommodations to match employee’s work with capacity and needs
5. Supervisor turns on support from OHP department and facilitates interventions on team or individual level

**Table 3:** Step 2: matrix with examples of change objectives per performance objective, based on the behavioural determinants

Performance objectives for supervisor	Logic of problem (from needs assessment)	Attitude [A] Social influence [S] Self-efficacy [E]	Skills, knowledge and self-regulation	Behaviour
**Change objectives for employees with CMHP to stay at work, the supervisor:**				
Talks with employee about the way mental health issues influence work	- Awareness raising on mental health	- Sees own role as crucial to support SAW [A]	- Able to observe and ask what employee needs	- Asks what employee needs to SAW
	- Balance between job demands and control	- Believes that employee with CMHP can work [A]	- Knows about interventions to offer	- Initiates dialogue by listening, and mirroring observations from work-related issue
	- Conversational skills training on mental health and work	- Sees how other supervisors support [S]	- Has conversational skills for sensitive topic	
	- Professional support from OHP	- Shows understanding, empathy [E]	- Encourages employee to share own solutions	
		- Has confidence to bring up work issues related to CMHP [E]	- Knows barriers on what (not) to ask	
Stimulates employee’s autonomy and sense of responsibility once CMHP influences work	- Employee’s experience of autonomy	- Believes in autonomy and responsibility by employee [A]	- Has skills to coach employee on a balanced sense of responsibility	- Stimulates employee to find own solutions but takes over when necessary
	- Active coping	- Knows how to stimulate employee to feel boundaries and say yes/no [E]	- Has knowledge on risk factors, signals	- Encourages employee to act on work-related and private issues
	- Information about roles and responsibilities	- Is confident that employee can and will take control [E]		
Supervisor talks with employee to match needs and capacity with work through job accommodations to SAW	- Ways to match employee’s capacities to work	- Is open to temporarily job accommodations to SAW (reduce/ change work / workplace) [A]	- Has knowledge about job accommodations and MH interventions	- Investigates with employee tasks, priorities and job accommodations
	- Supervisor’s knowledge and skills on interventions - Easy access and strong collaboration with OHP	- Knows boundaries on helping as supervisor and handover to OHP [S] - Is confident to find solutions with employee or gets support from OHP [E]		- Acts pro-actively on short term adjustments in work, besides giving space for interventions
	- Supervisor’s autonomy to apply tailored approach each employee	- Is confident to make exceptions so employee can SAW, explains accom-modations to team [E]		

*CMHP*   Common Mental Health Problem, *OHP* Occupational Health Professional, *SAW*  Stay At Work

### Step 3: core values, methods and practical strategies

The same stakeholder groups as in step 1 selected core values of the intervention, see Table 4. For example, that the intervention is practical, behaviour-oriented and can easily be used and delivered in various organizations. Also, participants emphasized that only providing an informative guideline is not enough to facilitate behavioural change. Ideas on types of interventions were psychoeducation through a guideline, tailored advice or consultation on individual case level and coaching on supervisor’s behaviour.

**Table 4:** Step 3: overall themes resulting in core values mentioned by stakeholders

Intervention should…
1) Address the theme in a socially safe climate and through openness on mental health problems
2) Define roles and responsibilities of supervisors
3) Be available as an online tool (interactive with links to websites) and hardcopy
4) Contain practical tips and tricks, to strengthen intention, skills and behaviour in various common situations
5) Tailor amount of information to the level of experience and needs of the supervisor, including a short version due to time constrains that supervisors often have, and avoiding jargon
6) Be easy to adopt, to access and deliver for organizations
7) Provide an overview with information on tools and basic conditions based on best practices and real-life dilemmas

Thereafter, methods and practical strategies were chosen to influence the change objectives, using the best available evidence. In this way, each behavioural determinant (attitude, social influence, self-efficacy, knowledge, skills, self-regulation and behaviour) is covered by one or two methods (Table 5). Literature on adult learning, health promoting behaviour and mechanisms of change was considered, see references in Table 5. The selection of practical strategies was based on the core values, technical options, feasibility, findings of the needs assessment and existing knowledge. For example, active transfer of information goal setting, guided practice and action learning in group can be applied. Some strategies can be performed by supervisors independently, such as studying the content of the guideline, or identifying cases among their team members. Other strategies need to be carried out by the implementers (OHPs) of the intervention, through consultation or coaching sessions individually or in small groups. For example, to identify and adjust beliefs towards mental health or to provide feedback on conversing skills.

**Table 5:** Step 3: selected theoretical methods and practical strategies for the determinants identified for the SAW-SG intervention

Determinant	Method	Practical strategy	Parameters for use by OHP or supervisor
**Intention**			
Attitude	- Belief selection [31] - Verbal persuasion [35, 38, 45]	- Identify current beliefs and strengthen positive beliefs and weaken negative beliefs, Introduce new beliefs	- Self-study or discussion with OHP individually or in group with other SVs
			- Select (un)supportive believes on CMHP and work—OHP leads sessions about GL by providing information, questions, arguments and dilemma’s
	- Modelling [46]	Identify role models Provide encouragement by stories and testimonials	- Mental health ambassadors discuss their work-related experiences with EM and SV in general
			- OHP speaks about success stories on how to SAW, possibly from within organization or videos
Social influence	- Social pressure [31]	Create sense of urgency on economic and societal impact Show success stories	- Movie with success stories in GL
			- OHP creates sense of urgency, shows numbers, risks on negative work outcomes, and examples
	- Social comparison among SV [47]	Provide opportunities for interaction among SV, Peer support groups	- Create support systems among SV about GL
			- OHP/HR department brainstorms or facilitates peer learning through intercollegial consultation
Self-efficacy	- Feedback [48]	Providing feedback Training and sharing of learned lessons among SV	- SV conducts self-study on GL, self-reflection
			- OHP advices SV per case about supportive behaviour, based on GL themes, in interactive sessions, consultation
			- OHP facilitates sessions in which SV introduces case and actions, in constructive feedback loops
	- Goals setting and action plans [49, 50]	Evaluation and action plans (if this, then I will…-plans)	- SV identify peer/coach to discuss
			- OHP coaches SV before dialogue with employee (if this, then I will…-plans), supported by GL
**Skills**			
Skills	- Guided practice [46]	Conversation checklist Guided practice Skills training on communication about MH	- SV identify peer/coach to receive coaching on skills development
			- Use of checklists in GL on conversational skills
			- Example movies or referral to other courses
			- OHP encourages SV to use reflection tools and GL
Knowledge	- Awareness raising [51] Discussion [38]	Evaluating understanding of magnitude of problem	- OHP and GL provides information about risks of absenteeism
			- OHP and SV discuss statistics of absenteeism in organization
			- OHP tailors information about organization
	- Active transfer of information [52]	Providing written and verbal information	- Information web tool/pdf about MH and role SV
			- Links to reliable external resources OHP shows and discusses content of GL with SV
Self-regulation and deal with barriers	- Feedback [48]	Define current approach, strengths and weaknesses Feedback on behaviour	- SV identify current approach, asks employees
			- OHP and SV identify solutions in GL for dilemmas in targets, internal processes that interfere with supporting SAW
**Behaviour**			
	- Goals setting and action plans [49, 50]	Diagram of actions Conversation checklist	- SV uses GL with diagram of actions to prepare
			- SV and OHP identify and evaluate goals and actions to increase employee’s MH
	- Tailoring [53]	Tailoring material to needs Consulting a professional (OHP)	- SV uses GL according to own needs and time
			- Organization facilitates regular and low-key opportunity to receive coaching to apply GL
	- Action learning in group [54]	Inter-collegial working groups Peer support through inter-collegial consultation	- SV in group discuss recent cases and their actions, advice each other on alternative actions or tips

*EM* Employee with CMHP, *SV* Supervisor, *OHP* Occupational Health Professional, *MH* Mental Health, *GL* Guideline

### Step 4: production and pre-test of intervention and materials

In this step, all gathered information from previous steps was synthesized to produce the intervention. Below, the scope and sequence of the SAW-SG intervention is presented, consisting of an online guideline and coaching sessions. Thereafter, findings of the pre-test are reported.

#### Online guideline

**Fig. 3:**
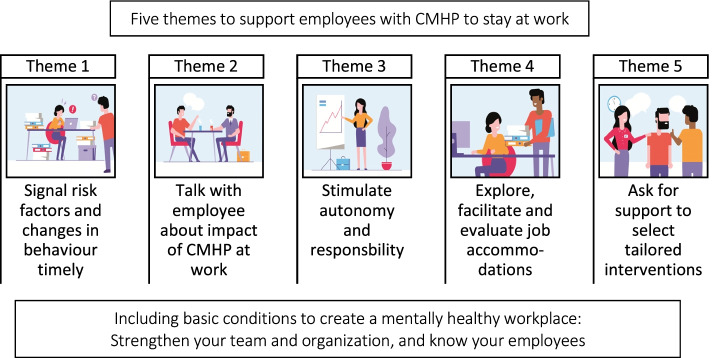
Step 4: overview of the Stay at Work Supervisor Guideline (SAW-SG) online version

The online guideline provides the supervisor with five step-wise themes on how to promote SAW (Fig. 3): 1) signal CMHP affecting the employee’s behaviour or work timely, 2) talk about impact of CMHP at work, 3) stimulate employee’s autonomy and sense of responsibility, 4) explore, facilitate and evaluate job accommodations to match work with employee’s needs and abilities, and 5) ask for occupational health support to select tailored interventions. Each theme is presented in three ‘layers’: from short and simple to long and more in-depth information, in order to tailor the amount and depth of information to the available time and needs of the supervisor. The first layer provides the most important actions presented in bullet points for supervisors, the second layer includes brief explanation and more specific actions, and the third layer offers the complete theme, including dilemma’s, checklists and additional information [53].

In general, the guideline stresses the dialogue between supervisor and employee. As shown in the needs assessment, much can be done by the supervisor in supporting employees who struggle with CMHP but are still at work. How persons talk about sensitive topics, such as their mental health, will depend much on the pre-existing relationship of employee and supervisor and the social climate in the work environment. Therefore, the guideline offers optional, supplementary information for supervisors to contribute to “mentally healthy workplaces”, concerning the environmental factors. This information is presented by two categories with basic conditions. The first category represents ways to know your employees in their regular work, e.g. on work values of employee, promoting a relationship of trust, attention for risk factors. Also, it addresses how to create a good fit between employee and their work and gives examples of interventions, both internal and external. The second category with basic conditions reflects ways to strengthen your team and organization on team-responsibility, safe working climate, social support among colleagues, mental health literacy and goals based on a shared vision on a ‘mentally healthy workplace’ and psychosocial work exposures, such as job strain.

Before the pre-test, the guideline was carefully reviewed as a member check to increase internal validity. The reviewers (editor on language, planning group and OHPs) appreciated the information presented in ‘layers’. They suggested to ensure the use of simple language so that supervisors supporting employees with a low socioeconomic position can also use the tools. Furthermore, they suggested to digitalize the guideline into an online product, a website, to increase accessibility and usability.

#### Interactive coaching sessions

Based on suggestions given by stakeholders and the selected methods and practical strategies, the interactive part of the intervention is drafted as follows. The guideline will be delivered through one plenary introduction session, followed by three monthly coaching sessions with supervisors, either individually or in small groups. Supervisors and OHPs mentioned that because it is a new approach, it is important to include other stakeholders within the organization in the process to create a supporting base on the organizational level, e.g. by inviting the HR professional during the introduction session. They also emphasized on the importance of delivering the intervention through the interactive coaching sessions. In those sessions, parameters for use (Table 5) are given, based on the needs assessment, chosen methods and strategies. For example, creating sense of urgency, identify possible solutions, advising supervisors per case and discussing dilemmas. These sessions were drafted around the content of the guideline, using input, such as cases or dilemmas brought up by supervisors to stimulate self-efficacy, skills and supportive behaviour. Ideally, these sessions were held by an independent OHP who has the following skills to apply the selected strategies: conversational skills on sensitive topics with both employers and employees and generally strong meta-communication skills such as non-judgemental listening, being patient and use of motivational interviewing. To ensure quality in those sessions, OHPs were trained on the guideline and coaching sessions before delivering the intervention, though a training based on the training protocol and training materials to facilitate the implementers.

#### Pre-test of the intervention

The prototype, a pdf version of the online guideline, was pre-tested by OHPs (*n* = 8) and supervisors (*n* = 7) on its usefulness, user-friendliness, and attractiveness. One supervisor dropped out due to time constraints. The pre-test was held fully online due to the COVID-19 restrictions in the autumn of 2020. The participants were positive about the guideline and found it useful, suitable and readable. Participants recognized the content of the guideline, its complexity and the practical information on actions to support. They also appreciated the ‘layered’ way of presenting information, however this could be improved using a website with more interactive and visual support. After the pre-test, the guideline was critically appraised and shortened by removing repetitions in text. Visual improvements were made on the online website to ease navigation and attractiveness. Also, more examples and actual workplace dilemmas were added. Lastly, as suggested by participants, an overview of all interventions available for employees to refer to was added.

As expected, participants confirmed that using the guideline by studying the five themes may increase intention (positive attitude towards mental health, social influence by feeling not alone in this, and self-efficacy since supervisors gain insight into their supportive behaviour through the guideline), however improvements in skills and actual supportive behaviour occurs through training and coaching. Therefore, participants particularly appreciated the interactive coaching sessions, in which they discussed the most applicable tools and actions in their particular situation and they were challenged to reflect upon their behaviour. They also suggested to invite someone with lived experience of CMHP, to share experience talking *with* employees rather than talking only *about* employees with CMHP. Therefore, we included this in the training of OHPs and during the introduction session with supervisors, by inviting employees who are experts by lived experience, from advocacy organizations.

OHPs mentioned that the training protocol offered them clear instructions on how to introduce the guideline, but at the same time they valued professional flexibility to adjust the selected strategies and training material in the interactive sessions to their own organization. They also found the selected strategies useful, for example to identify current beliefs and to set and evaluate goals through feedback.

### Step 5. Planning for adoption and implementation

In the fifth step, a plan for the adoption and implementation of the intervention was developed. The following requirements were identified for optimal adoption of the intervention. First, higher management of the participating organization should support the implementation of the SAW-SG. Second, representatives in organisations regarding occupational health services and human resources need to recognize the urgency of the problem (by high numbers of absenteeism) and need to be motivated to a novel intervention. Third, it seemed beneficial that the OHP and the particular representative in the organization have yet established a working relationship, which can help for example to identify supervisors as participants. Lastly, it is important that organizations receive clear and concise information about the process and content of the intervention, especially the benefits, costs and amount of time it takes for all involved stakeholders.

The experiences with the SAW-SG intervention will be assessed in an implementation and evaluation study in 2021. We aim to include approximately 20 OHPs for implementing the SAW-SG intervention, delivering the intervention to 3–6 supervisors per organization. Participants agreed on the following success factors for delivery of the intervention: that supervisors 1) are facilitated to spend at least 5 h to this intervention spread over 3 months, 2) are interested in such a project and 3) have recently or currently had at least one employee with mental health issues in their team, in order to practice during the intervention.

### Step 6. Planning for evaluation

In the sixth and final step of the IM process, an evaluation design was chosen including a plan for the evaluation of the impact as well as the implementation process. To evaluate this intervention, we will use a realist evaluation approach answering the research question: what works (or not), for whom, under what circumstances and how [55]? We choose this theory-driven evaluation approach because implementation of interventions at the workplace highly varies as to how organizational support and occupational health services are organized (the circumstances), as well as the variety of implementation strategies (how does it work) between stakeholders on individual, interpersonal and organizational levels (and for whom). The forthcoming evaluation study will present results on the following aspects: the process of implementation, the mechanisms of change and contextual factors, leading to the intended and unintended outcomes.

## Discussion

This study presents the development of the Stay at work-Supervisor Guideline (SAW-SG) intervention to strengthen supervisor support, promoting employees with CMHP to stay at work. Development of the intervention was guided by the IM approach, which resulted in an online guideline and a training protocol for interactive coaching sessions to support supervisors. The online guideline contains five themes to signal and address problems in the workplace and find solutions by stimulating the employee’s autonomy, explore job accommodations and ask for occupational health support. Labour experts as OHPs delivered the intervention as they are independent, and experts in matching employee’s capabilities with work and work environment.

The SAW-SG intervention adds to the literature on workplace interventions in mental health, through an innovative, evidence-based intervention with a preventive approach by strengthening the supervisor's supportive behaviour regarding mental health at work. In line with these previous IM studies, we endorse that (individual) behavioural models on employee-level can be transferred to the behaviour of other workplace stakeholders as individuals who act as change agents in an organization. The additional value of the Integrated model of behaviour prediction was the integrative approach towards behaviour, in which environmental and general motivational factors also were included in the intervention, both content wise by the included basic conditions in the guideline and for delivery through the implementation strategies. In this, the intervention targets the complexity between individual behaviour and actions, and the interaction, often on a interpersonal level, with the work context. Although it is challenging to realize changes in organizational culture or support systems, this study made a first step by facilitating change on the interpersonal level by improving the interaction between OHP, supervisor and employee [34]. Nevertheless, we did not specifically target psychosocial work exposures that significantly associated with mental health outcomes, as revealed in a recent meta-analysis [56]. Reflecting on our intervention, these were indirectly addressed in the basic conditions (job strain, psychological demands) and in theme 3 and 4 respectively (stimulating autonomy of employees to avoid decision latitude and explore job accommodations to adjust long working hours).

Staying at work, for employees with CMHP, is a relatively new concept, that is not clearly defined in the literature [5]. This implies also that ways to promote stay at work are not yet profoundly developed and evaluated in the literature. Therefore, a considerable amount of time was needed to identify promoting factors to SAW for which we used both theory and practice during the needs assessment. Theory of working mechanisms to stay at work on both employee-level and organization-level were retrieved by a systematic realist literature review [5]. In addition, these promoting factors to stay at work for employees and the role of the supervisor were verified in practice with various workplace stakeholders through a concept mapping study [57] and focus group discussions. Altogether, this provided content to the intervention, including practical ways to support employees with CMHP who struggle at work. As a result, this study adds to the conceptualization of staying at work.

This intervention turned out to target three key areas, namely general awareness on mental health, basic conditions for a mentally healthy workplace and five stepwise themes with actions to support employees with CMHP. In its essence, these all reflect the way supervisors do position and treat employees with CMHP. Promoting a trustful relationship between supervisor and employee, both before and whilst struggling at work due to mental health problems, was highlighted by all participants as a main challenge for supervisors. In this, the dialogue between employee and supervisor is an important element to signal and talk about symptoms in an early stage. Supervisors addressed the necessity of such an intervention to train all supervisors addressing ‘soft skills’, possibly mandatory, contributing to the quality of this dialogue [39]. As found in other studies, they need to be facilitated by their organisation, through individual coaching and peer learning through consultation among colleague-supervisors [22, 32]. It underscores the growing realization by employers that they should and can act pro-actively in prevention to promote mental health at the workplace, by being given the appropriate guidance [58].

Non-surprisingly, many of the actions and themes addressed in the guideline seemed relevant to all employees: those with and without CMHP. All participants in our study stressed the early signalling and addressing of work-related issues, in a phase that mental health problems are present but not (yet) lead to sick leave. There is a thin line, especially in prevention, between addressing mental health *in general* and addressing mental health *problems that affect one’s work.* Thus, it can be argued that our intervention does not only benefit employees with CMHP but all employees, possibly resulting into more trustful and sustainable working relationships. We observed during our study that investing in awareness and skills among supervisors leads to more attention and empathy for mental wellbeing of employees in general. Also, basic conditions to create mentally healthy workplaces were addressed, that may reduce psychosocial work exposures that associate with negative health outcomes [56]. Another study found that this may eventually create more disclosure about mental health issues at the workplace leading to adequate supervisor support [24].

The SAW-SG intervention was tailored to the rather new role of labour experts as OHPs in the Dutch context, shifting their services in return to work trajectories towards prevention. Various workplace stakeholders in our study appreciated the role of these implementers. Reasons were that they are being trained to match employee’s needs with the work functioning and work environment, being independent, pragmatic and familiar with the work environment, as suggested by the literature in the needs assessment [5]. However, selecting labour experts as OHPs to deliver this intervention has its limitations. Firstly, the recruitment of labour experts in this study showed that especially those who feel competent to offer psycho-education and coaching are interested to deliver such an intervention. This is a relatively small group having these skills due to various educational backgrounds before these professionals join their training for labour expert. Secondly, many organizations do not have access to a labour expert as OHP. This may limit the broader, nationwide dissemination of the intervention and its sustainability. Thirdly, in various other countries, the role of labour experts and other OHPs differs from the Dutch setting. Therefore, we believe that other OHPs such as organizational psychologists, HR managers who are trained in prevention and mental health or occupational health nurses could also deliver the intervention.

### Methodological considerations

Intervention mapping was considered as a valuable tool as it provided a systematic process to identify, structure and prioritize factors and select practical strategies to induce the targeted behaviour. Our initial idea was to develop a guideline, offering information to employers on how to promote SAW for employees with CMHP. However, the evidence gathered in the IM steps and a rigor, theory-based approach, led to the insight that such a guideline can only be effective when delivered through interactive sessions. Therefore, we elaborated the intervention. Although we followed the IM procedure stepwise, we reflected on previous steps also, which led to more optimal use of the input from participants. For example, when reducing the content of the online guideline after the pre-test (step 4), we moved back to the needs assessment to reprioritize the changeable factors.

Especially employers indicated that they need an intervention that can be tailored and easily accessed. IM has been helpful to ensure that despite the plethora of factors to promote SAW, the intervention resulted into a manageable and accessible amount of information. Also, the IM approach helped the researchers to actively and early involve a broad range of stakeholders, that is often aimed by researchers but hard to realize in practice. Paying particularly attention to the participative planning group and workplace stakeholders in each step led to strong adherence and commitment throughout the process [41]. OHP and employers were actively involved throughout the IM-process, resulting in an intervention that is well-received. Representatives of employees with CMHP were actively involved, however, we could not collect data on employee-level during the pre-test, due to privacy regulations and sensitivity to disclose CMHP. It would have been better to investigate the perception of employees with CMHP, as done in a previous study by Bjork Brämberg et al. [58]. This also applies to the implementation and evaluation phase, as we target the behaviour and behavioural determinants of supervisors as a direct, proximal outcome of the intervention. Due to the given reasons above and due to various external factors resulting in employee’s well-being or perception of supervisor support, we choose not to evaluate on those outcomes.

Among both supervisors and OHPs, there was some ambivalence regarding the delivery and adherence of the guideline and training protocol, in which on one hand participants appreciated the specific tools and actions on how to support employees with CMHP. On the other hand, they emphasized on their professional flexibility, especially to consider and weigh actively the suggested actions versus the specific case, stimulating a critical attitude towards their own behaviour. Therefore, we decided to present actions in the guideline as options and facilitate feedback and discussion through the interactive coaching sessions. Likewise, we provided suggestions for training material and practical strategies for OHPs, but left room for adjustments. Permitting this level of flexibility in intervention delivery and adherence is somehow contrary to the IM approach, that provides a structured way to monitor and ensure the delivery of the intervention as intended [59]. As a result, there may be a difference between the suggested tools and actions and the actual supportive behaviour. Thereby, the pilot implementation and evaluation study can provide more insights on the use of the guideline, and what worked, under what circumstances, how and why.

### Future research and practical implications

Although the IM process was valuable, it does not guarantee for success [41, 59]. The forthcoming implementation study will lead to information about the process and impact of the SAW-SG intervention, including the feasibility of selected outcome measures. This will inform researchers and professionals how the intervention can be imbedded in organizations and in educational programs for labour experts and other OHPs. Resulting from this study, we suggest that, through the IM approach or other approaches, researchers and program developers should actively involve multiple stakeholders throughout the process, on a basis of partnership. Ideally, both implementers, users (e.g. supervisors) and ultimate beneficiaries should be involved from as early as possible until evaluation and dissemination.

In such intervention development it is hard to grasp what actually happens during delivery, in line with our choice to allow professional flexibility in intervention delivery for both OHPs and supervisors to tailor information according to their needs [59]. In future research, we suggest to investigate in practice which strategies have been used during the implementation phase and what the effect was of each. Namely, each strategy can be considered a micro intervention, in which different working mechanisms may be triggered in specific circumstances, leading to intended or unintended outcomes. To better understand those, we recommend to use alternative paradigms to the use of RCTs to bring novel insights into the conditions of their implementation, impact and generalization of the intervention, such as realist evaluation [41, 55, 60].

The presented intervention targets mainly organizations in which there is a rather traditional ‘supervisor-employee’ relationship based on a rather traditional type of employment in which the line manager is the representative of the formal employer of the employee who has an employment contract. Participants in this study mentioned that the intervention may not (yet) be suitable for more modern, upcoming, types of employment, such as temporary employment agencies, secondment agencies and self-managing teams. Also, we reached mainly large-sized companies and struggled to include medium-small sized companies. Those diversities in employment types may require different implementation strategies or further development of the current guideline.

## Conclusions

This study describes how the ‘SAW-Supervisor Guideline’ intervention was developed to strengthen supervisor support, resulting in an online guideline and interactive coaching sessions. The guideline addresses five themes on how to promote SAW while employees with CMHP struggle at work, based on the best available evidence. Also, it proved the importance of the dialogue between employee and supervisor, before and while struggling at work due to mental health issues, based on a trustful relationship. This intervention seems promising as it responds to the needs of supervisors in their role, responsibility and ways to support employees with mental health issues, through a behaviour-oriented, preventative approach. Supervisors learn how to signal and address mental health issues and match work and the working context with capabilities of employees. Intervention mapping provided a systematic process to identify, structure and prioritize goals and elements on how to promote SAW. The active involvement of workplace stakeholders throughout the process led to a well-received intervention with feasible implementation strategies. The Integrated model of behaviour prediction provided insights into novel, practical ways to induce the targeted behaviour of workplace stakeholders, bridging theory with practice. The results of the realist impact evaluation on this intervention will be available in 2022.

## Data Availability

The datasets generated and/or analyzed during the current study are available from the corresponding author on reasonable request.

## Abbreviations

SAW: Stay at work
CMHP: Common mental health problems
IM: Intervention mapping
OHP: Occupational health professional
SAW-SG: Stay at work supervisor guideline
